# Mechanisms of Infection in Zika Virus

**DOI:** 10.3390/pathogens12081035

**Published:** 2023-08-14

**Authors:** Ana Belén Blázquez

**Affiliations:** Department of Biotechnology, Instituto Nacional de Investigación y Tecnología Agraria y Alimentaria (INIA-CSIC), 28040 Madrid, Spain; blazquez@inia.csic.es

Zika virus (ZIKV) is a mosquito-borne flavivirus that was first identified in Uganda in 1947, then was essentially neglected for six decades. However, in 2007, a ZIKV outbreak emerged in the Western Pacific Ocean, and between 2013 and 2014, a larger outbreak spread in the South Pacific, reaching South America in 2015 [1]. In 2016, the World Health Organization (WHO) declared a Public Health Emergency of International Concern. Even though the virus is transmitted by mosquitoes, mainly of the genus *Aedes* (*Stegomyia*), other transmission routes have been reported: between sexual partners and from mother to fetus, as well as via the transfusion of blood and blood products [2]. 

ZIKV infection usually causes a self-limited disease presenting acute febrile illness, but recent studies link the infection to a rising number of severe neurologic complications in adults, such as Guillain–Barré syndrome, myelitis, and meningoencephalitis, as well as congenital ZIKV infection that can result in Zika congenital syndrome including fetal damage and microcephaly in newborns [3]. 

Despite efforts to find one, there is currently no commercially available vaccine or treatment to prevent or fight against this viral pathogen [4,5], resulting in an urgent need for research into the mechanisms of ZIKV infection.

As we have learnt from the recent pandemic caused by SARS-CoV-2, we cannot predict what might come next or when that might be, but it is clear that viruses can emerge unexpectedly and cause global human disease. All the challenges in, and possible contributions to, unveiling the pathogenic mechanism of ZIKV are welcome for the current Special Issue entitled “Mechanisms of Infection in Zika Virus”, which aims to analyze and discuss the development of diagnostic methods, prevention and control strategies, and genetic mechanism of the virus, provide solutions, and expand the knowledge covering all aspects of viral pathogenesis and host–virus interactions, essential steps in creating new strategies to fight the virus. 
